# Higher serum resistin levels and increased frailty risk in older adults: Implications beyond metabolic function

**DOI:** 10.1016/j.jnha.2025.100521

**Published:** 2025-02-20

**Authors:** Beom-Jun Kim, Yunju Jo, Ji Yeon Baek, So Jeong Park, Hee-Won Jung, Eunju Lee, Il-Young Jang, Hyuk Sakong, Dongryeol Ryu

**Affiliations:** a Division of Endocrinology and Metabolism, Department of Internal Medicine, Asan Medical Center, University of Ulsan College of Medicine, Seoul 05505, South Korea; b Department of Biomedical Science and Engineering, Gwangju Institute of Science and Technology, Gwangju 61005, South Korea; c Division of Geriatrics, Department of Internal Medicine, Asan Medical Center, University of Ulsan College of Medicine, Seoul 05505, South Korea; d Asan Institute for Life Sciences, Asan Medical Center, University of Ulsan College of Medicine, Seoul 05505, South Korea

**Keywords:** Resistin, Frailty, Aging, Biomarker, Adipokine

## Abstract

**Background:**

Despite the pleiotropic role of resistin as an adipokine, its association with frailty—an indicator of biologic age and overall well-being in humans—remains largely unexplored. This study aims to investigate the potential of circulating resistin as a biomarker for frailty.

**Methods:**

The study included 228 older adults aged 65 years or older who underwent a comprehensive geriatric assessment. Frailty was evaluated using both the phenotypic frailty model by Fried and the deficit-accumulation frailty index (FI) by Rockwood. Serum resistin levels were measured using a competitive enzyme-linked immunosorbent assay.

**Results:**

After adjusting for sex, age, body mass index, smoking, alcohol, exercise, diabetes, and serum creatinine, serum resistin levels were 52.2% higher in individuals with phenotypic frailty than in robust controls (*P* =  0.001) and showed a positive correlation with the Rockwood FI (*P* =  0.015). Furthermore, for every 1 standard deviation increase in serum resistin levels, the risk of frailty increased by 67% (*P* =  0.021). When participants were divided into four groups based on serum resistin levels, individuals in the highest quartile had a 38% higher FI and exhibited a 12.5-fold higher odds ratio for frailty compared to those in the lowest quartile (*P* =  0.016 and 0.024, respectively).

**Conclusion:**

These findings suggest that circulating resistin may serve as a candidate blood-based biomarker for frailty, encompassing the multifaceted physical, cognitive, and social dimensions, extending beyond its well-established role in metabolic regulation.

## 1 Introduction

In an era characterized by a rapidly aging population, the concept of frailty has emerged as a paramount concern in public health, given its well-documented links to adverse outcomes including functional decline, increased healthcare utilization, and elevated mortality rates [1,2]. The assessment of frailty predominantly relies on two widely recognized methodologies: the "phenotypic frailty" approach and the "frailty index" [3]. Each of these assessment tools presents unique features and applications in clinical practice and research settings. The phenotypic frailty model employs a set of observable clinical indicators, encompassing factors such as unintentional weight loss, subjective fatigue, and decreased physical activity levels [4]. This approach offers clinicians a practical and efficient means of identifying individuals at risk of frailty. In contrast, the frailty index adopts a more comprehensive stance, utilizing a cumulative deficit framework that incorporates a wide array of health-related variables, including coexisting medical conditions, functional limitations, and abnormal laboratory parameters [5,6]. While both methodologies have demonstrated efficacy in frailty assessment, they each possess distinct strengths and limitations. The phenotypic model's simplicity facilitates its implementation in various clinical environments, whereas the frailty index provides a more detailed and nuanced evaluation of an individual's overall health status [3,7]. Given their complementary nature, the integration of both approaches in biomarker research could significantly enhance the identification of high-risk frail populations and inform strategies aimed at promoting healthy aging.

Resistin, originally identified as an adipokine implicated in insulin resistance and glucose metabolism by regulating inflammation [8,9], has emerged as a multifaceted factor with far-reaching implications for human health. Initially associated with obesity and metabolic syndrome, recent evidence has expanded its role to encompass a diverse array of pathological conditions, including cardiovascular diseases, liver disorders, respiratory ailments, infectious diseases, and renal complications [8,[10], [11], [12]]. This pleiotropic nature suggests that resistin may exert a comprehensive influence on overall health status. As a secreted protein abundantly present in human circulation and readily quantifiable, resistin holds promise as a potential biomarker for various age-related conditions, including dementia, sarcopenia, and osteoporosis [[13], [14], [15]]. Despite the growing interest in resistin's systemic effects beyond metabolic function, its relationship with frailty—a clinical syndrome reflecting an individual's biological age and overall well-being—remains largely unexplored. This study aims to address this gap by investigating the association between circulating resistin levels and frailty in a cohort of older adults, using both the phenotypic frailty model and the frailty index to provide a comprehensive assessment of frailty status and evaluate resistin's potential as a biomarker for this condition."

## 2 Materials and methods

### 2.1 Study population and design

This clinical investigation enrolled older adults aged 65 and above from Korea who underwent a comprehensive geriatric assessment (CGA) at the Division of Endocrinology or Geriatrics, Department of Internal Medicine, Asan Medical Center (AMC) in Seoul, South Korea, between April 2019 and January 2021. Participants sought medical attention for non-specific symptoms common in older adults, such as fatigue and decreased appetite, or for management of chronic conditions including osteoarthritis, hypertension, and hyperlipidemia. All subjects were ambulatory and community-dwelling, not residing in institutional settings. The study excluded individuals with end-stage renal disease, active malignancy, or symptomatic heart failure with an expected survival of less than one year. Blood samples were obtained from 228 eligible participants who provided informed consent after exclusion of ineligible individuals (Supplementary Fig. S1). The AMC Institutional Review Board approved this study (approval no. 2020-0259), which adhered to the ethical principles outlined in the Declaration of Helsinki.

### 2.2 Comprehensive geriatric assessment protocol

Experienced nurses conducted the CGA, gathering data through in-depth interviews and medical record reviews. The assessment collected information on demographic details, medical history, and prior surgical procedures. It utilized previously validated CGA-frailty index variables [16], encompassing geriatric domains such as comorbidities, functional capacity, physical performance, nutritional status, and common geriatric syndromes including cognitive impairment, depression, and polypharmacy. Multimorbidity was defined as having two or more of the 18 physician-diagnosed conditions including hypertension, stroke, peripheral vascular disease, myocardial infarction, heart failure, coronary artery disease, atrial fibrillation/flutter, angina, diabetes, depression, sensory deficits, degenerative spinal disease, cancer in the past five years, chronic kidney disease (estimated glomerular filtration rate <60), chronic obstructive pulmonary disease, asthma, arthritis, and anxiety disorders. Disability was defined as requiring assistance with any of the seven activities of daily living (ADLs) or with any of the seven instrumental ADLs (IADLs).

### 2.3 Frailty assessment methodologies

#### 2.3.1 Phenotypic frailty evaluation

Frailty was assessed using the Cardiovascular Health Study frailty criteria, a widely accepted model developed by Fried et al. [4]. This model evaluates five key components: unintended weight loss, slowness, weakness, low physical activity, and self-reported exhaustion. These components were scored, and participants were classified into robust (0 points), prefrail (1–2 points), or frail (3–5 points) categories based on their total score.

#### 2.3.2 Deficit-accumulation frailty index

The frailty index, as proposed by Rockwood et al. [5], was employed as a sensitive predictor of adverse outcomes. It evaluates the cumulative impact of psychosocial, medical, and functional deficits related to aging. In this study, a frailty index was computed, validated in previous research (see Supplementary Table S1 for full list of items) [16,17]. The frailty index score is calculated by dividing the number of identified deficits by 49 evaluable items, with scores ranging from 0 to 1. Higher scores indicate greater frailty.

### 2.4 Body composition and functional Status assessment

Body composition, including muscle and fat mass, was measured using a bioelectrical impedance analyzer (InBody S10; InBody, Seoul, Korea), which operates at frequencies of 1, 5, 50, 250, 500, and 1000 kHz [18]. To minimize the effects of recent food or fluid intake, participants fasted for at least 8 h before the test. Appendicular skeletal muscle mass (ASM) was calculated by adding the muscle mass of the arms and legs, and the skeletal muscle index (SMI) was determined by normalizing ASM to the square of the participant’s height, allowing for objective comparisons. Handgrip strength on the dominant hand was measured using the Jamar hydraulic hand dynamometer (Patterson Medical, Warrenville, IL, USA) [19]. Participants performed the test while seated, with elbows at a 90-degree angle, exerting maximal force. Two measurements were taken, separated by at least one minute, and the highest value was recorded. Gait speed was assessed over a 4-meter distance, and the time for completing five chair stands was recorded. The Short Physical Performance Battery (SPPB) was used to evaluate lower extremity function, incorporating repeated chair stands, balance tests, and gait speed measurements. Balance was assessed in three stances: side-by-side, semi-tandem, and tandem, with participants maintaining each position for up to 10 seconds. The SPPB score ranged from 0 to 12, with higher scores indicating better physical performance.

### 2.5 Resistin measurement protocol

Blood samples were collected after at least 8 h of fasting. Following centrifugation at 3,000 rpm for 5 min at 4 °C, the serum was separated and stored at −80 °C for subsequent analysis. Hemolyzed or clotting samples were discarded. Serum resistin levels were quantified using a competitive enzyme-linked immunosorbent assay (ELISA) kit (Cat. No. DRSN00; R&D Systems, Minneapolis, MN) following the manufacturer's instructions. The assay's detection limit was 0.055 ng/mL, with intra-assay and inter-assay coefficients of variation below 5.3% and 9.2%, respectively.

### 2.6 Statistical analysis approach

All data are presented as means ± standard deviation (SD) or as frequencies and percentages, unless specified otherwise. Comparisons of baseline characteristics among groups based on phenotypic frailty status were performed using analysis of variance (ANOVA) with post-hoc Tukey's test for continuous variables and the χ² test for categorical variables. To adjust for potential confounders, analysis of covariance (ANCOVA) was used to calculate and compare estimated means with 95% confidence intervals for serum resistin levels according to phenotypic frailty status and for the frailty index according to serum resistin quartiles. The confounding variables, including sex, age, body mass index (BMI), smoking, regular alcohol consumption, regular exercise, diabetes, and serum creatinine, were selected based on their significant association with serum resistin in univariate analysis or their clinical relevance. Regular alcohol consumption was defined as consuming alcohol at least once per week on average, and regular exercise was defined as engaging in moderate-intensity physical activity—characterized by a slight increase in breathing—at least three times per week. Pearson’s correlation and partial correlation analyses were applied to assess the relationship between frailty-related factors and serum resistin levels. Logistic regression was utilized to calculate odds ratios (ORs) for phenotypic frailty status relative to increasing serum resistin levels and quartiles. Statistical analyses were performed using SPSS version 18.0 (SPSS Inc., Chicago, IL, USA), with significance defined as *P* <  0.05.

## 3 Results

Table 1 delineates the clinical characteristics of 228 participants in the study. Applying the Fried criteria, 79 (34.6%) were classified as robust, 123 (54.0%) as prefrail, and 26 (11.4%) as frail. The proportion of females in each group was 65 (82.3%), 103 (83.7%), and 19 (73.1%), respectively (*P* =  0.436). The mean age for robust, prefrail, and frail groups was 74.2 ± 5.1, 76.3 ± 5.4, and 80.9 ± 6.0 years, respectively (*P* <  0.001). Compared to the robust or prefrail groups, older adults in the frail group were characterized by shorter stature, weaker grip strength, slower gait speed, lower SPPB scores, and reduced muscle mass (*P* <  0.001 to 0.024). Furthermore, frail individuals exhibited higher serum creatinine levels and Rockwood frailty index scores, longer chair stand test times, and significantly higher prevalence of sarcopenia, polypharmacy (≥5 medications), multimorbidity, and disability (*P* <  0.001 to 0.031). However, weight, BMI, lifestyle factors, and serum albumin levels did not differ significantly among the three groups.

**Table 1:** Clinical characteristics of study participants according to phenotypic frailty status.

	Robust	Prefrail	Frail	
Variables	(N = 79)	(N = 123)	(N = 26)	*P* value
Sex, no (%)				0.436
Male	14 (17.7)	20 (16.3)	7 (26.9)	
Female	65 (82.3)	103 (83.7)	19 (73.1)	
Age (years)	**74.2 ± 5.1**	76.3 ± 5.4*	80.9 ± 6.0^*,#^	**<0.001**
Weight (kg)	56.7 ± 8.6	57.9 ± 10.7	53.5 ± 8.5	0.119
Height (cm)	**155.5 ± 6.3**	**153.7 ± 6.9**	150.4 ± 6.8*	**0.004**
BMI (kg/m^2^)	23.4 3.0	24.6 ± 3.9	23.7 ± 3.3	0.068
Serum albumin (g/dL)	3.92 ± 0.23	3.90 ± 0.25	3.82 ± 0.26	0.180
Serum creatinine (mg/dL)	**0.80 ± 0.17**	**0.85 ± 0.34**	0.96 ± 0.21*	**0.031**
Smoking, no (%)	11 (13.9)	18 (14.6)	3 (11.5)	0.918
Regular alcohol consumption, no (%)	6 (7.6)	9 (7.3)	2 (7.7)	0.996
Regular exercise, no (%)	13 (16.5)	16 (13.0)	0 (0.0)	0.091
Diabetes, no (%)	27 (34.2)	45 (36.6)	13 (50.0)	0.341
Frailty index (range, 0−1)	**0.054 ± 0.037**	0.108 ± 0.069*	0.236 ± 0.109^*,#^	**<0.001**
Grip strength (kg)	**26.6 ± 6.9**	23.9 ± 5.8*	18.3 ± 5.9^*,#^	**<0.001**
Gait speed (m/s)	**1.15 ± 0.15**	0.92 ± 0.23*	0.64 ± 0.20^*,#^	**<0.001**
Chair stand (s)	**9.2 ± 2.6**	12.4 ± 8.8*	19.4 ± 15.2^*,#^	**<0.001**
SPPB score (range, 0−12)	**11.6 ± 0.8**	10.3 ± 1.9*	7.3 ± 2.8^*,#^	**<0.001**
ASM (kg)	**15.0 ± 3.0**	**14.4 ± 2.9**	12.9 ± 2.7*	**0.007**
SMI (kg/m^2^)	**6.17 ± 0.86**	**6.04 ± 0.84**	5.64 ± 0.79*	**0.024**
Sarcopenia, no (%)	**2 (2.5)**	**34 (27.6)**	**20 (76.9)**	**<0.001**
Use of ≥5 prescription of drugs, no (%)	**29 (36.7)**	**68 (55.3)**	**22 (84.6)**	**<0.001**
Multimorbidity, no (%)	**45 (57.0)**	**87 (70.7)**	**23 (88.5)**	**0.007**
ADL disability, no (%)	**3 (3.8)**	**6 (4.9)**	**8 (30.8)**	**<0.001**
IADL disability, no (%)	**3 (3.8)**	**15 (12.2)**	**17 (65.4)**	**<0.001**

*P* values were analyzed by ANOVA for continuous variables or χ^2^ test for categorical variables. ^*,#^Statistically significant difference from the robust and prefrail groups, respectively, by post hoc analysis using Tukey’s method. Bold indicates that values are statistically significant. BMI, body mass index; SPPB, short physical performance battery; ASM, appendicular skeletal muscle mass; SMI, skeletal muscle index; ADL, activities of daily living; IADL, instrumental activities of daily living.

**Fig. 1:**
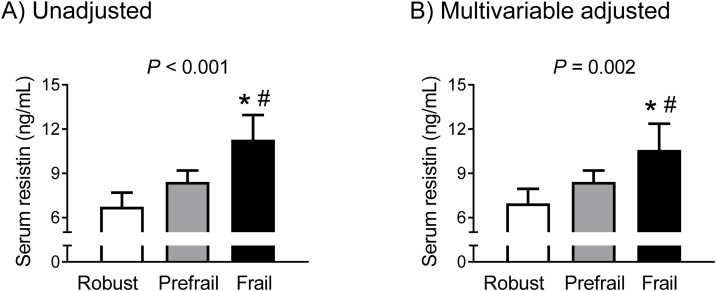
Differences in serum resistin levels according to phenotypic frailty status (A) before and (B) after adjusting for potential confounders. The multivariable adjustment model includes sex, age, BMI, smoking, alcohol, exercise, diabetes, and serum creatinine. The estimated means with 95% confidence intervals were generated and compared using an analysis of covariance. ^*,#^Statistically significant difference from the robust and prefrail groups, respectively. BMI, body mass index. Phenotypic frailty is defined based on Fried’s criteria.

Analysis of circulating resistin concentrations according to phenotypic frailty status using ANCOVA revealed that in the unadjusted analysis, the frail group had 67.7% and 34.0% higher levels compared to the robust and prefrail groups, respectively (*P* <  0.001 and *P* =  0.002, respectively) (Fig. 1A). These differences remained statistically significant after adjusting for sex, age, BMI, smoking, alcohol, exercise, diabetes, and serum creatinine (*P* =  0.001 and *P* =  0.027, respectively) (Fig. 1B)

Both before and after adjusting for confounding variables, serum resistin concentrations showed a positive correlation with the Rockwood frailty index (*P* <  0.001 and 0.015, respectively) (Table 2). Regarding physical function indicators, after adjusting for sex, age, BMI, smoking, alcohol, exercise, diabetes, and serum creatinine, serum resistin demonstrated negative correlations with gait speed, SPPB score, and SMI, while showing a positive correlation with chair stand test completion time (*P* =  0.012 to 0.027). However, no statistical significance was observed with grip strength.

**Table 2:** Association between serum resistin levels and frailty-related parameters.

Frailty-related parameters	Unadjusted		Multivariable adjusted	
	γ *	*P* value	γ ^†^	*P* value
Frailty index	**0.254**	**<0.001**	**0.163**	**0.015**
Grip strength	–0.124	0.061	–0.110	0.105
Gait speed	**–0.231**	**<0.001**	**–0.156**	**0.021**
Chair stand	**0.197**	**0.003**	**0.157**	**0.019**
SPPB score	**–0.233**	**<0.001**	**–0.149**	**0.027**
SMI	–0.092	0.165	**–0.169**	**0.012**

The multivariable adjustment model includes sex, age, BMI, smoking, alcohol, exercise, diabetes, and serum creatinine. Bold indicates that values are statistically significant. SPPB, short physical performance battery; SMI, skeletal muscle mass index; BMI, body mass index. *Pearson’s correlation coefficient; ^†^Partial correlation coefficient.

The risk of frailty associated with increased serum resistin levels was analyzed using logistic regression analyses (Table 3). Before adjusting for confounding variables, the OR for frailty was 2.02 for each SD increase in serum resistin (*P* <  0.001). This statistical significance persisted with an OR of 1.67 after adjusting for sex, age, BMI, smoking, alcohol, exercise, diabetes, and serum creatinine (*P* =  0.021)

**Table 3:** Odds ratios for phenotypic frailty according to the increase in serum resistin.

Model	ORs per SD increments in serum resistin	95% CI	*P* value
Unadjusted	**2.02**	**1.37–2.97**	**<0.001**
Multivariable adjusted	**1.67**	**1.08–2.56**	**0.021**

*P* values were analyzed by logistic regression analysis. The multivariable adjustment model includes sex, age, BMI, smoking, alcohol, exercise, diabetes, and serum creatinine. Bold indicates that values are statistically significant. BMI, body mass index; ORs: odds ratios, SD; standard deviation; CI, confidence interval. Phenotypic frailty is defined based on the Fried’s criteria.

**Fig. 2:**
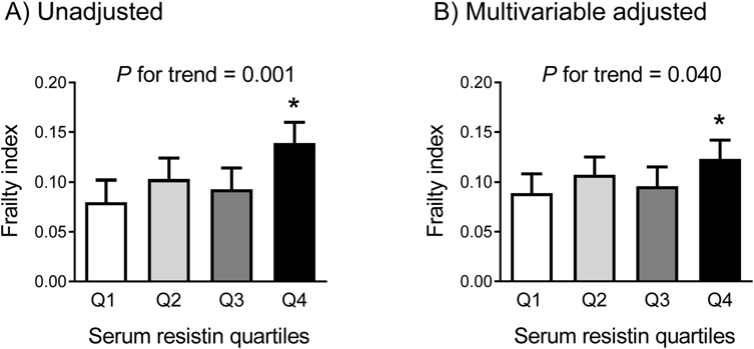
Differences in the frailty index according to serum resistin quartiles (A) before and (B) after adjusting for potential confounders. The multivariable adjustment model includes sex, age, BMI, smoking, alcohol, exercise, diabetes, and serum creatinine. The estimated means with 95% confidence intervals were generated and compared using an analysis of covariance. *Statistically significant difference from the lowest quartile (Q1). BMI, body mass index. The frailty index is calculated based on Rockwood’s proposal. Serum resistin quartiles: Q1 = 1.72–4.80 ng/mL; Q2 = 4.81–6.80 ng/mL; Q3 = 6.81–10.70 ng/mL; Q4 = 10.71–21.02 ng/mL.

To investigate a potential threshold effect between circulating resistin concentrations and the Rockwood frailty index, participants were categorized into four groups based on resistin levels. Compared to individuals in the lowest resistin quartile (Q1), those in the highest quartile (Q4) were older, had slower gait speed, lower SPPB scores, and a higher prevalence of sarcopenia and polypharmacy (*P =* 0.002 to 0.049; Supplementary Table S2). Notably, an increasing trend in the frailty index was observed across resistin quartiles, regardless of the adjustment model, with individuals in the Q4 having at least a 38% higher frailty index compared to those in the Q1 (*P* =  0.001 to 0.016; Fig. 2). Additionally, logistic regression analysis revealed that individuals in the Q4 had 16.5-fold higher OR before adjusting for confounding variables (*P* =  0.008), and 12.5-fold higher OR after multivariable adjustment for frailty (*P* =  0.024) (Table 4).

**Table 4:** Odds ratios for phenotypic frailty according to serum resistin quartiles.

Resistin quartiles	Unadjusted		Multivariable adjusted	
	Odds ratio (95% CIs)	*P*	Odds ratio (95% CIs)	*P*
Q1	1 (reference)		1 (reference)	
Q2	7.84 (0.93–66.0)	0.058	9.47 (1.02–89.4)	0.052
Q3	5.39 (0.61–47.6)	0.130	5.27 (0.52–53.0)	0.159
Q4	**16.5 (2.08–131.4)**	**0.008**	**12.5 (1.39–112.2)**	**0.024**

*P* values were analyzed by logistic regression analysis. The multivariable adjustment model includes sex, age, BMI, smoking, alcohol, exercise, diabetes, and serum creatinine. Bold indicates that values are statistically significant BMI, body mass index; CI, confidence interval. Phenotypic frailty is defined based on Fried’s criteria. Serum resistin quartiles: Q1 = 1.72–4.80 ng/mL; Q2 = 4.81–6.80 ng/mL; Q3 = 6.81–10.70 ng/mL; Q4 = 10.71–21.02 ng/mL.

## 4 Discussion

Our investigation, conducted in a cohort including older adults aged 65 and above, revealed a significant elevation in serum resistin concentrations among those classified as frail, even after adjustment for potential confounders. Furthermore, we observed a positive correlation between increasing levels of circulating resistin and both the frailty index score and the likelihood of meeting criteria for phenotypic frailty. To our knowledge, this research represents the inaugural clinical study exploring the link between frailty and resistin levels in the bloodstream. The results carry substantial clinical relevance, suggesting that resistin may serve as a candidate for a blood-based biomarker of frailty encompassing the multifaceted physical, cognitive, and social dimensions, extending beyond its well-established role in metabolic regulation.

Resistin, derived from the term "resistance to insulin," was initially identified as a cytokine secreted by adipocytes in rodents, with its primary function linked to metabolic processes such as insulin resistance and inflammation [8,9]. In humans, resistin is predominantly produced by monocytes and macrophages [20] and is associated with various diseases, including cardiovascular conditions, respiratory and liver diseases, and cancers [[21], [22], [23], [24]]. Frailty, a clinical syndrome characterized by declines in physical, cognitive, and social functioning, serves as an integrative measure of biological age, reflecting an individual's overall health status more accurately than chronological age [1,3,25]. Given resistin's involvement in diverse pathophysiological processes and its role as a secreted protein, it has the potential to act as a circulating biomarker for frailty in older adults. In our study, the observed association between serum resistin levels and frailty underscores its clinical significance, providing a foundation for future research to investigate resistin as a predictive marker for frailty and a target for interventions to improve health outcomes in older adults.

Higher circulating resistin levels are associated with an increased risk of frailty in older adults through several interconnected mechanisms. First, resistin promotes the production of pro-inflammatory cytokines, such as TNF-α, IL-6, and IL-1β, leading to chronic low-grade inflammation or "inflammaging" [8,26], which accelerates muscle protein degradation and impairs tissue repair and regeneration [27,28]. Additionally, resistin contributes to insulin resistance, which not only reduces muscle glucose uptake but also disrupts anabolic signaling pathways like IGF-1/Akt/mTOR, essential for muscle protein synthesis [[29], [30], [31], [32]]. This metabolic dysfunction is further compounded by oxidative stress, as resistin induces the production of reactive oxygen species (ROS) and compromises mitochondrial function [33,34], resulting in muscle fatigue, diminished endurance, and reduced physical performance. Moreover, resistin reflects adipose tissue dysfunction, particularly in visceral fat, leading to systemic lipotoxicity that infiltrates muscle tissue and impairs muscle contractility and quality [35,36]. Emerging evidence also suggests that resistin influences bone metabolism, disrupting the bone-muscle crosstalk crucial for maintaining musculoskeletal integrity and increasing the risk of osteoporosis and fractures [[37], [38], [39]], both key components of frailty. Finally, resistin is linked to reduced physical activity levels due to systemic inflammation and metabolic strain [40,41], creating a vicious cycle where lower activity exacerbates muscle deconditioning and further amplifies frailty progression. Collectively, these mechanisms highlight the multifactorial role of resistin in impairing muscle mass, strength, and physical performance, underscoring its potential contribution to the development and progression of frailty in older adults.

A key strength of our study lies in the application of both the phenotypic frailty model and the Rockwood frailty index, emphasizing the complementary value of these two widely recognized assessment tools. The phenotypic frailty model, derived from the Fried criteria, is extensively used in clinical and research contexts due to its straightforward application, time efficiency, and focus on physical components, enabling quick assessments [4]. In contrast, the Rockwood frailty index incorporates a wider spectrum of deficits, including cognitive, psychological, and social domains, making it a more holistic measure [5,6]. Although its administration is more time-intensive, it provides a stronger correlation with adverse outcomes such as hospitalization, disability, and mortality and offers a more accurate reflection of biological age [7,25,42]. By employing both methods, our study provides a novel perspective on the relationship between frailty and resistin. This integrated approach enhances the robustness and comprehensiveness of our findings, setting our research apart in the field of frailty biomarker studies.

Our study has several limitations that warrant consideration. First, the cross-sectional design prevents the establishment of a causal relationship between serum resistin levels and frailty, making it uncertain whether resistin actively contributes to the development of frailty or merely serves as a bystander. Second, our cohort lacks information on inflammatory markers such as TNF-α or IL-6, preventing us from evaluating whether the hypothesized adverse effects of resistin on human health are mediated through these factors. Third, due to the challenges of simultaneously obtaining data for both the Rockwood frailty index and the Fried phenotype model, along with collecting blood samples from older adults, the number of enrolled participants who provided consent was relatively small. However, we believe that this preliminary analysis provides an important foundation for future studies. Fourth, the study population consisted exclusively of Korean individuals, which restricts the generalizability of the results to other ethnic groups. Lastly, we cannot rule out the possibility that our results were influenced by various factors that may affect circulating resistin levels or frailty in humans.

In conclusion, this study in a cohort of ambulatory, community-dwelling older adults demonstrated that serum resistin levels were significantly higher in those with phenotypic frailty and were positively correlated with the frailty index. As frailty serves as a critical marker of overall health and functional capacity, these findings suggest that resistin may play a broader role in human homeostasis beyond its established metabolic function. Large-scale longitudinal studies are needed to further evaluate the potential of circulating resistin as a predictive blood biomarker for the onset and progression of frailty.

## Funding

This research was supported by grants from the Korean ARPA-H Project through the Korea Health Industry Development Institute (KHIDI), funded by the Ministry of Health and Welfare, Republic of Korea [Grant Number: RS-2024-00507256]; the Korea Health Technology R&D Project through KHIDI, also funded by the Ministry of Health and Welfare, Republic of Korea [Grant Number: RS-2024-00401934]; the National Research Foundation of Korea (NRF), funded by the South Korean government (MSIT) [Grant Number: RS-2022-NR074857]; and the Asan Institute for Life Sciences, Asan Medical Center, Seoul, Republic of Korea [Grant Number: 2024IL0013].

## Data availability

The data that support the findings of this study are available from the corresponding author by investigators with institutional review board approval.

## Declaration of competing interest

The authors declare that they have no known competing financial interests or personal relationships that could have appeared to influence the work reported in this paper.
