# Supplementary material for: Higher serum resistin levels and increased frailty risk in older adults: Implications beyond metabolic function
Source: J Nutr Health Aging. 2025 Feb 20;29(5):100521. doi: 10.1016/j.jnha.2025.100521 (PMC12180029; doi:10.1016/j.jnha.2025.100521)
Supplement: Supplementary file 1 [file mmc1.docx]

**Supplementary Table 1.** Items for comprehensive geriatric assessment-frailty index

| **Medical History (21 items)** | | | |
| --- | --- | --- | --- |
| • Angina  • Anxiety disorder  • Arthritis  • Asthma  • Atrial fibrillation/flutter  • Cancer within 5 years  • Chronic kidney disease (eGFR < 60) | • COPD  • Coronary artery disease  • Degenerative spine disease  • Dementia  • Depression  • Diabetes  • Fall within the past year | | • Heart failure  • Hypertension  • Myocardial infarction  • Peripheral vascular disease  • Sensory impairment  • Stroke/TIA  • Use of ≥ 5 prescription drugs |
| **Functional Status (21 items)** | | | |
| **Activities of Daily Living**  • Feeding  • Dressing/undressing  • Grooming  • Walking (or use of a walker)  • Getting in and out of bed  • Toileting  • Bathing or shower | **Activities of Daily Living**  • Using telephone  • Using transportation  • Shopping  • Preparing own meals  • Housework  • Taking own medications  • Managing money | | **Nagi and Rosow-Breslau Activities**  • Pulling or pushing a large object  • Stooping, crouching or kneeling  • Lifting or carrying 10 lbs  • Reaching arms above shoulder  • Writing or handling small objects  • Walking up/down a flight of stairs  • Heavy work around house |
| **Performance Tests (4 items)** | | | |
| **Mini-Mental Status Examination**  27–30 points (0 points)  24–26 points (0.3 points)  21–23 points (0.7 points)  <21 points (1 point) | | **5 Repeated Chair Stands**  <11.20 s (0 points)  11.20–13.69 s (0.25 points)  13.70–16.69 s (0.5 points)  16.70–60.9 s (0.75 points)  ≥ 61.0 s (1 point) | |
| **Gait Speed**  ≥ 1 m/sec (0 points)  0.80–0.99 m/s (0.3 points)  0.60–0.79 m/sec (0.7 points)  <0.60 m/s (1 point) | | **Dominant Handgrip Strength**  M, ≥ 32 kg; F, ≥ 20 kg (0 points)  M, ≥ 26–31 kg; F, 16–19 kg (0.5 points)  M, <26 kg; F, <16 kg (1 point) | |
| **Nutritional Status (3 items)** | |  | |
| • Weight loss > 4.5 kg in past year | • Body mass index < 21 kg/m^2^ | | • Serum albumin < 3.5 g/dL |

Abbreviations: COPD, chronic obstructive pulmonary disease; eGFR, estimated glomerular filtration rate; F, female; M, male; TIA, transient ischemic attack.

**Supplementary Table 2.** Clinical characteristics of study participants according to serum resistin quartiles

| Variables | Serum resistin quartiles | | | | *P* value |
| --- | --- | --- | --- | --- | --- |
|  | Q1 (N = 57) | Q2 (N = 57) | Q3 (N = 57) | Q4 (N = 57) |  |
| Sex, no (%) |  |  |  |  | 0.625 |
| Male | 8 (14.0) | 13 (22.8) | 9 (15.8) | 11 (19.3) |  |
| Female | 49 (86.0) | 44 (77.2) | 48 (84.2) | 46 (80.7) |  |
| Age (years) | **74.9 ± 5.7** | **75.7 ± 5.2** | **76.2 ± 5.9** | **77.8 ± 5.8^*^** | **0.049** |
| Weight (kg) | 58.0 ± 8.8 | 58.2 ± 9.5 | 54.8 ± 10.4 | 56.8 ± 10.4 | 0.209 |
| Height (cm) | 154.8 ± 6.4 | 155.3 ± 6.5 | 152.4 ± 7.0 | 153.2 ± 7.2 | 0.088 |
| BMI (kg/m^2^) | 24.2 ± 3.0 | 24.2 ± 3.9 | 23.6 ± 3.9 | 24.4 ± 3.5 | 0.612 |
| Fall within the past year, no (%) | 12 (21.1) | 8 (14.0) | 9 (15.8) | 12 (21.1) | 0.678 |
| Hypertension, no (%) | 34 (59.6) | 30 (52.6) | 32 (56.1) | 39 (68.4) | 0.355 |
| Diabetes, no (%) | 22 (38.6) | 21 (36.8) | 17 (29.8) | 25 (43.9) | 0.483 |
| Grip strength (kg) | 24.7 ± 7.1 | 24.7 ± 6.4 | 24.9 ± 7.2 | 22.6 ± 5.8 | 0.211 |
| Gait speed (m/s) | **1.01 ± 0.19** | **1.02 ± 0.26** | **0.99 ± 0.28** | **0.87 ± 0.25^*^** | **0.004** |
| Chair stand (s) | 10.5 ± 3.4 | 12.1 ± 8.2 | 11.3 ± 7.5 | 14.4 ± 13.2 | 0.095 |
| SPPB score (range, 0-12) | **11.1 ± 1.5** | **10.4 ± 2.1** | **10.7 ± 1.9** | **9.7 ± 2.8^*^** | **0.004** |
| ASM (kg) | 14.8 ± 3.0 | 14.9 ± 2.8 | 14.0 ± 3.4 | 14.0 ± 2.8 | 0.171 |
| SMI (kg/m^2^) | 6.13 ± 0.79 | 6.15 ± 0.82 | 5.94 ± 1.02 | 5.92 ± 0.74 | 0.314 |
| Sarcopenia, no (%) | **9 (15.8)** | **12 (21.1)** | **13 (22.8)** | **22 (38.6)** | **0.031** |
| Use of ≥5 prescription of drugs, no (%) | **23 (40.4)** | **33 (57.9)** | **23 (40.4)** | **40 (70.2)** | **0.002** |
| Multimorbidity, no (%) | 39 (68.4) | 40 (70.2) | 32 (56.1) | 44 (77.2) | 0.110 |

*P* values were analyzed by ANOVA for continuous variables or χ^2^ test for categorical variables. *Statistically significant difference from the lowest quartile (Q1) by post hoc analysis using Tukey’s method. Bold indicates that values are statistically significant. BMI, body mass index; SPPB, short physical performance battery; ASM, appendicular skeletal muscle mass; SMI, skeletal muscle index. Serum resistin quartiles: Q1 = 1.72–4.80 ng/mL; Q2 = 4.81–6.80 ng/mL; Q3 = 6.81–10.70 ng/mL; Q4 = 10.71–21.02 ng/mL.
