# Supplementary material for: Higher serum resistin levels and increased frailty risk in older adults: Implications beyond metabolic function
Source: J Nutr Health Aging. 2025 Feb 20;29(5):100521. doi: 10.1016/j.jnha.2025.100521 (PMC12180029; doi:10.1016/j.jnha.2025.100521)
Supplement: Supplementary file 2 [file mmc2.docx]

**
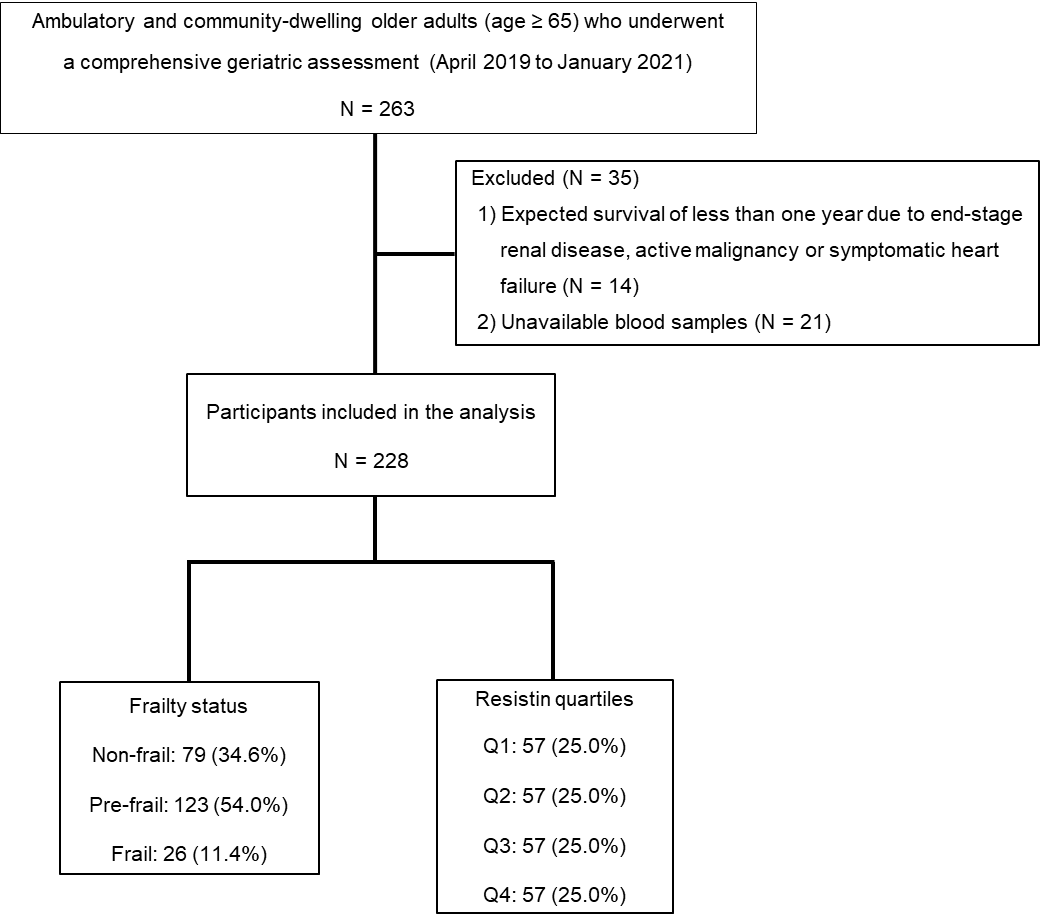
**

**Supplementary Figure 1.** Flow Diagram of Study Participants. Q, quartile. Serum resistin quartiles: Q1 = 1.72–4.80 ng/mL; Q2 = 4.81–6.80 ng/mL; Q3 = 6.81–10.70 ng/mL; Q4 = 10.71–21.02 ng/mL.
